# Iron Compartmentalisation and Vascular Endothelial Cell Dysfunction

**DOI:** 10.3390/antiox15060757

**Published:** 2026-06-15

**Authors:** Theo Issitt, George W. Kagugbe, Quezia K. Toe, S. John Wort, Gregory J. Quinlan

**Affiliations:** 1 National Heart and Lung Institute, Faculty of Medicine, Imperial College London, London SW3 6L, UK; theoissitt@gmail.com (T.I.); g.kagugube@ucl.ac.uk (G.W.K.); q.toe15@imperial.ac.uk (Q.K.T.); s.wort@nhs.net (S.J.W.); 2 Organic and Biological Analytical Chemistry Group (OBIACHEM), MolSys Research Unit, Liège University, B-4000 Liege, Belgium; 3 Faculty of Life Sciences, Division of Biosciences, University College London, Gower Street, London WC1E 6BT, UK; 4 Royal Brompton Hospital, Adult Centre for Pulmonary Hypertension, London SW3 6NP, UK

**Keywords:** iron compartmentalisation, vascular endothelial cells, pulmonary hypertension, hepcidin–ferroportin axis, ferroptosis, reactive oxygen species, haemolysis, mitochondrial dysfunction, non-transferrin-bound iron, pulmonary vascular remodelling

## Abstract

Iron is essential for life, but its safe use by the body depends on it being kept within tightly controlled compartments. When this compartmentalisation is disrupted—through haemolysis, saturation of scavenger proteins, or dysregulation of the hepcidin–ferroportin axis—damaging iron species accumulate in the circulation and within vascular cells, with potentially serious consequences for endothelial function. This review explores the mechanisms by which iron dysregulation compromises vascular endothelial cell biology across a range of disease states, including haemolytic anaemias, atherosclerosis, cerebrovascular disease, extracorporeal circulatory support, and iatrogenic iron loading. Common pathological themes emerge: depletion of nitric oxide bioavailability, oxidative stress, endothelial activation, and in chronic settings, vascular remodelling. The review subsequently focuses in depth on the pulmonary vasculature, where dysregulated iron compartmentalisation has emerged as a key contributor to the pathogenesis of pulmonary hypertension. Here, iron-driven mitochondrial dysfunction, smooth muscle cell proliferation, and iron-dependent lipid peroxidation via ferroptosis are discussed as mechanistic drivers of pulmonary vascular remodelling. The therapeutic implications of targeting iron handling in pulmonary hypertension are considered, including modulation of the hepcidin–ferroportin axis. Together, the evidence presented highlights disordered iron compartmentalisation as a unifying pathological thread across vascular disease and a compelling target for intervention.

## Simple Summary

Iron is essential for aerobic life, yet its safe biological deployment depends entirely on strict compartmentalisation within protective protein environments. This review examines how the disruption of iron compartmentalisation—whether through haemolysis, saturation of scavenger proteins, dysregulation of the hepcidin–ferroportin axis, or intracellular iron mishandling—compromises vascular endothelial cell function across a broad range of disease settings. We consider the mechanisms by which free haem, cell-free haemoglobin, and non-transferrin-bound iron gain access to the vascular endothelium, and the downstream consequences for nitric oxide bioavailability, oxidative stress, endothelial activation, and vascular remodelling. The review then focuses on pulmonary hypertension as a disease in which dysregulated iron compartmentalisation is increasingly recognised as a key pathogenic driver, exploring its influence on mitochondrial function, smooth muscle cell proliferation, and ferroptosis in pulmonary vascular cells. Finally, the therapeutic implications of targeting iron handling in pulmonary hypertension are considered. Together, the evidence presented positions disordered iron compartmentalisation as a unifying pathological mechanism across vascular disease and a compelling, if complex, target for intervention.

## 1. Introduction

**Figure 1:**
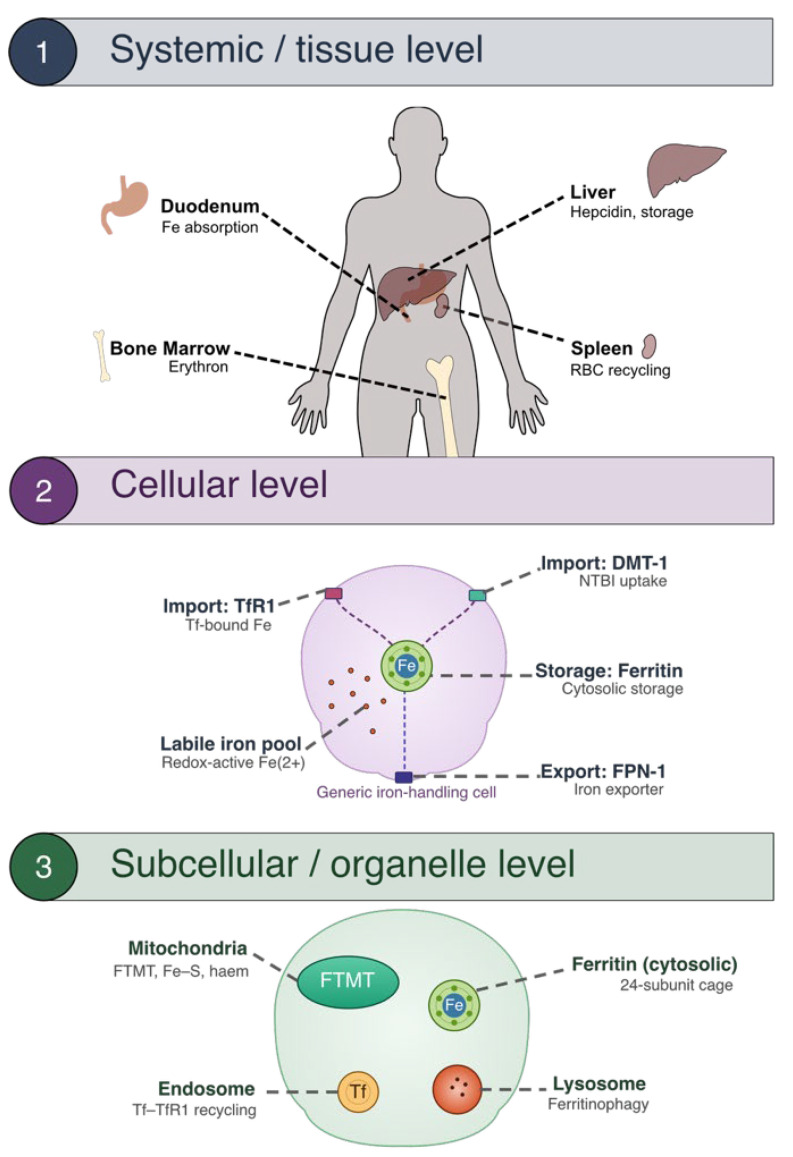
Hierarchical levels of iron compartmentalisation in health. Iron is segregated across three regulatory levels. (1) Systemic/tissue level. Body iron is partitioned between the circulation (transferrin-bound iron), the erythron and bone marrow (haematopoiesis; ~2500 mg, the largest pool), the duodenum (the sole site of dietary absorption, 1–2 mg/day), and the principal storage and recycling organs—the liver (hepatic ferritin) and the spleen (macrophage recycling of senescent erythrocytes). At this level, iron flux is governed principally by the hepcidin–ferroportin axis: hepatic hepcidin senses circulating iron, inflammation (IL-6), and erythropoietic demand, and restricts cellular iron efflux by binding and degrading the iron exporter ferroportin (FPN-1). (2) Cellular level. Within individual cells, iron import (transferrin receptor 1, TfR1; divalent metal transporter 1, DMT-1), cytosolic storage (ferritin) and export (FPN-1) are balanced around a small, redox-active labile iron pool. Coordination is achieved post-transcriptionally through the iron regulatory protein/iron-responsive element (IRP/IRE) system: under iron deficiency, IRPs bind 5′ IREs to repress the translation of ferritin and ferroportin, and 3′ IREs to stabilise TfR1 and DMT-1 transcripts, increasing uptake. (3) Subcellular/organelle level. Within the cell, iron is further sequestered into discrete compartments: cytosolic ferritin (a 24-subunit cage holding up to ~4500 Fe atoms in redox-silent ferric form), mitochondria (haem and Fe–S cluster biosynthesis, buffered by mitochondrial ferritin, FTMT), the endosomal system (transferrin–TfR1 recycling), and lysosomes (ferritinophagy and autophagic iron turnover). Compartment-specific handling proteins (mitoferrins, frataxin, NCOA4, TRPML1) coordinate flux between these compartments and prevent the participation of iron in Fenton chemistry. Loss of regulatory control at any of these levels constitutes iron decompartmentalisation, resulting in the expansion of labile redox-active iron pools and increased susceptibility to iron-catalysed oxidative injury, the downstream consequences of which for vascular endothelium and pulmonary vascular disease are the subject of Figure 3.

Iron is an essential trace metal in human physiology, with a total body content of roughly 2–4 g in adults [1]. In the broadest terms, iron facilitates aerobic metabolism as many of the major physiological functions of iron are linked to oxygen utilisation. Notable examples include respiration, transport, storage, antioxidant protection, and biosynthesis. As a variable valence transition metal with the ability to donate or accept single electrons between the ferrous (Fe^2+^) and ferric (Fe^3+^) states through single-electron transfer reactions (and in some cases higher oxidation states), iron can activate oxygen from a relatively inert ground state to more reactive and metabolically functional intermediates. The electronic structure of ground state molecular oxygen provides inherent stability to this molecule due to the presence of unpaired electrons with parallel spin in the outermost orbitals of each oxygen atom. This so-called spin restriction limits the ability of oxygen to disrupt covalent bonds, as electron pairs within covalent bonds exist in antiparallel pairs. Ground state molecular oxygen is therefore largely inert, even though in chemical terms, it is an oxygen free radical. The ability of iron ions to overcome this spin restriction renders oxygen derivatives reactive and metabolically functional. Effective iron utilisation is largely facilitated via the localisation of iron in active centres of protein/enzyme systems within which iron catalysis is strictly controlled and regulated, including within haem centres and iron sulphur clusters [2]. In this review, iron compartmentalisation denotes the segregation of iron into defined, protein-bounded environments across three hierarchical levels, as shown in Figure 1: (i) the systemic or tissue level, at which iron is partitioned between the circulation, the erythron, and storage organs such as the liver and spleen, and is governed principally by the hepcidin–ferroportin axis; (ii) the cellular level, at which import, storage, and export are balanced largely through IRP/IRE-mediated post-transcriptional control; and (iii) the subcellular or organelle level, at which iron is sequestered within ferritin, mitochondria, and lysosomes. Loss of regulatory control at any of these levels constitutes a breakdown of compartmentalisation and is the unifying theme of this review. Oxygen transport and storage are similarly rendered safe via the compartmentalisation of iron-containing moieties. However, if iron is not adequately constrained within such protective environments, unrestrained iron-catalysed reactive oxygen species (ROS) production can ensue, resulting in significant oxidative stress, and in more extreme circumstances, oxidative damage to biomolecules and tissues, particularly once endogenous primary antioxidant mechanisms become overwhelmed [3]. In this context, the principal extracellular antioxidant defence is provided by the iron- and haem-binding proteins of plasma—including transferrin, haptoglobin, haemopexin, albumin, and ceruloplasmin [4,5,6,7]—which collectively neutralise the redox capacity of iron species at their active binding sites, preventing catalytic ROS generation rather than simply scavenging reactive species once formed.

In mammals, iron is principally obtained from the gut, mainly via the duodenal epithelium with uptake equating to 1–2 mg/day (adult male). However, daily iron requirements, principally for haem biosynthesis to facilitate the production of 200 billion red cells per day, are 10–20 mg/day [8]. Uptake from the gut cannot satisfy this requirement; therefore, to compensate for this deficit, body iron resources are largely recycled via erythrophagocytosis of senescent red blood cells by specialised macrophages of the spleen and liver, which together account for approximately 90% of daily iron needs [9,10]. Importantly, in this regard, there is no known regulated mechanism for body iron excretion; instead, losses occur via exfoliation of intestinal epithelium, shedding of skin cells, and blood loss, together with some loss due to the excretion of a non-reabsorbed fraction of bile and urine [8]. Such losses are offset mainly by gut uptake. The resident iron pool is therefore a valuable but limited resource that requires careful management. The need to recycle iron requires mobilisation and shunting between compartments; all such actions are tightly controlled to limit the risk of adverse catalytic effects (damaging ROS formation), preserve a limited and valuable resource, and restrict the availability of iron as a growth factor for microbial virulence. Bacteria utilise various means of sequestering iron from host organisms, ranging in degree of sophistication from simple siderophores (bacterial-derived iron-binding agents) to the expression of transferrin-like receptors and other receptor mimics [11,12]. Limiting iron availability to microbial pathogens remains a lifelong challenge for the host immune system, particularly in ageing and chronic disease states. Other consequences of disrupted iron homeostasis include altered cell-fate decisions, ranging from inappropriate proliferative responses to ferroptotic cell death. Iron is a requirement for DNA biosynthesis via ribonucleotide reductase and for the action of several cell cycle cyclins, playing a central role in the regulation of ferroptoic cell death [13]. The shunting and compartmentalisation of iron beyond cellular storage capacity trigger such events in cells not typically associated with iron turnover and control. Consequently, balancing the body’s iron requirements with potential adverse consequences is a physiological challenge under normal circumstances. However, in states of tissue injury or inflammation, where elevated ROS production occurs alongside the potential for microbial invasion, the need for iron management becomes even more acute. Therefore, very tight control of mobilisation and utilisation is required in both health and disease.

## 2. Regulation and Homeostatic Control of Iron Resources

### 2.1. Iron Regulatory Proteins (IRPs)

**Figure 2:**
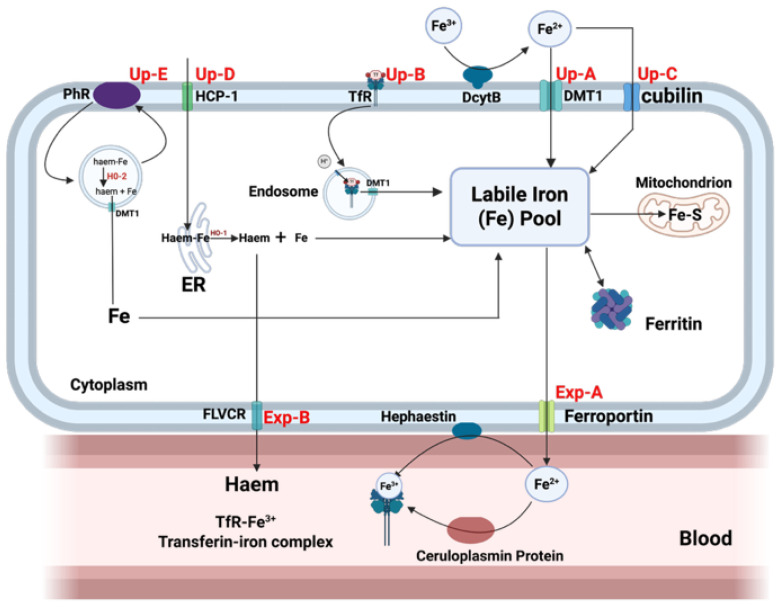
Cellular iron homeostasis: uptake, storage and export. **Up-A**, Central to the uptake of iron from the diet is the duodenal cytochrome b-like ferrireductase (DcytB), which reduces ferric (Fe^3+^) iron to ferrous (Fe^2+^) that is readily taken up by the divalent metal transporter 1 (DMT-1) into the cell [14,15]. DMT-1 is also involved in the endosomal release of iron after endocytosis of the transferrin (Tf)-transferrin receptor (TfR) complex into cells. **Up-B** refers to transferrin receptor (TfR)-mediated endocytic iron uptake. The main route of cellular iron uptake is via the transferrin receptors 1 (TfR1) and 2 (TfR2). In this uptake mechanism, the entire Tf-Fe complex bound to TfR is endocytosed, forming an intracellular endosome. Iron is released following protonation (via the H^+^ pump) of the endosome, which causes a conformational change in the Tf-Fe complex, thereby releasing endosomal iron via DMT-1. Both apo-transferrin and TfR are predominantly recycled back to the cell surface, although a fraction may undergo lysosomal degradation [23]. TfR1 is ubiquitously expressed throughout iron-handling cells, but TfR2 is present only in hepatocytes and erythrocyte progenitor cells, where it serves an iron-sensing role [15,24]. Megalin-cubilin-mediated uptake has been demonstrated in the kidney proximal tubule, where it may contribute to iron reabsorption, particularly under conditions of iron overload or reduced TfR1 expression. In the kidneys, a multi-ligand heteromeric receptor complex, megalin/cubilin **Up-C,** has been shown to transport iron into proximal tubules, particularly when TfR1 is severely reduced, even during proximal tubule iron overload [16]. This supports the hypothesis that iron reabsorption through epithelial cells in the distal and proximal tubules of the kidneys lowers iron loss via kidney excretion [25,26]. Haem iron uptake mainly occurs in macrophages, hepatocytes, and duodenal enterocytes. It involves direct uptake through haem carrier protein 1 (HCP-1) **Up-E** and putative haem receptor (PhR) mediated endocytosis **Up-D**. Endocytosed haem is subsequently degraded by haem oxygenases, releasing iron into the cytosolic pool. HO-2 is constitutively expressed, whereas HO-1 is inducible and commonly upregulated in response to haem exposure and oxidative stress. Once inside cells, the fate of iron depends on the type of cell and its iron load state. For example, enterocytes will immediately release the iron into the circulation, where it is transported bound to the plasma iron transporter transferrin (Tf). In other cells, such as hepatocytes, astrocytes, and macrophages, iron is often sequestered and stored in ferritin or used for the biosynthesis of iron-sulphur clusters (Fe-S) and cytochromes within mitochondria [1,15]. If cellular iron export is required, Fe^2+^ is exported via ferroportin 1 (FPN-1) **Exp-A**. On cellular export, Fe^2+^ is rapidly converted to Fe^3+^through the ferroxidase activity of membrane-bound hephaestin, or the plasma proteins ceruloplasmin (Cp) and its glycosylphosphatidylinositol (GPI)-anchored membrane isoform. In the ferric state, iron can be safely bound to Tf for transport or lactoferrin from neutrophils at lower pH. Haem export is facilitated by feline leukaemia virus subgroup C (FLVCR) **Exp-B**. Both hemopexin and albumin bind and transport haem in blood. Haptoglobin binds and transports free haemoglobin in an analogous manner. Importantly, by sequestering iron and haem at their binding sites, these plasma proteins contribute primary antioxidant protection—neutralising the redox reactivity of iron species before catalytic ROS generation can occur [27], in addition to their transport function [6,17,28,29].

Body iron content is regulated at both the cellular (local) and systemic (global) levels. Local control mechanisms regulate influx, storage, and efflux by controlling cellular proteins involved in iron homeostasis and metabolism. A major mechanism regulating cellular control is the post-translational control of mRNAs encoding proteins involved in iron homeostasis [14,15,16,17]. Iron regulatory proteins (IRPs) are well-documented as molecular ON/OFF switches through binding to stem loop motifs known as iron responsive elements (IREs) found in the 5′ and 3′ non-coding sections of mRNA [18,19]. Under conditions of cellular iron deficiency, IRPs bind with high affinity to IREs. Binding to a 5′ IRE, located in the 5′ untranslated region, sterically blocks ribosomal recruitment and represses (OFF-switch) translation of the target mRNA into protein [18,20,21]. The principal 5′ IRE-containing transcripts encode the iron-storage protein ferritin (H- and L-subunits) and the iron exporter ferroportin (FPN-1); their repression therefore limits iron storage and export when iron is scarce. Conversely, when IRPs bind to 3′ IREs, such as those in the transferrin receptor 1 (TfR1) and certain divalent metal transporter 1 (DMT-1) transcripts, they stabilise these mRNAs against nuclease degradation, prolonging transcript survival and enhancing cellular iron uptake (ON-switch) under iron-deficient conditions (Figure 2). When cellular iron is replete, IRP IRE-binding activity is lost: ferritin and ferroportin are translated, whereas TfR1 and DMT-1 transcripts are degraded, so that iron uptake falls and iron storage increases. Beyond canonical iron sensing, nitric oxide can directly modulate this system: nitrosative disruption of Fe–S clusters promotes conversion of the bifunctional cytosolic aconitase to its IRP1 form, thereby altering ferritin translation, iron uptake and intracellular iron storage dynamics [18,22].

### 2.2. The Hepcidin-Ferroportin Axis

At the systemic level, the primary regulator of iron homeostasis and metabolism is the 25-amino acid peptide hormone hepcidin. First isolated and described as an antimicrobial peptide identified in the liver, hepcidin is now recognised as the primary regulator of global iron homeostasis [30].

As for the mode of action, hepcidin targets ferroportin (FPN), the only known cellular iron exporter [31], leading to endocytosis and lysosomal degradation, thereby preventing cellular iron export [21,32]. The actions of hepcidin, therefore, serve to lower plasma iron levels, whereas its suppression increases plasma iron levels, with opposing effects on the cellular environment. The gene coding for hepcidin (HAMP) is downregulated by erythropoiesis, anaemia, and hypoxia, thereby increasing the plasma iron concentration by increasing cellular iron export through ferroportin [33,34,35,36]. Iron overload and inflammatory cytokines, particularly interleukin 6 (IL-6) and interleukin-1 beta (IL-1β), increase the expression of HAMP and therefore increase hepcidin [37]. Various iron- and haem-iron-mediated mechanisms for cellular iron uptake have been identified (see Figure 2 and the legend for a detailed explanation). To date, ferroportin remains the only established cellular exporter of non-haem iron. The hepcidin–ferroportin axis is therefore central to systemic iron homeostasis and cellular iron availability. Ferroportin is prominently expressed in enterocytes, where it facilitates and controls the release of dietary iron into the blood circulation. Additionally, cells classically responsible for iron turnover and recycling including hepatocytes, Kupffer cells, subtypes of macrophages and erythroblasts also express high levels of ferroportin. Whilst most research to date has focused on these essential drivers of iron turnover and control, it is important to recognise that all cells have iron requirements, and the recognition of the presence of ferroportin in other cell types including vascular endothelial cells is now established [38].

### 2.3. Hypoxia-Inducible Factors (HIFs)

HIFs play a central role in coupling oxygen sensing to iron homeostasis. Under low-oxygen or iron conditions, HIF-α subunits are stabilised (because the iron-dependent PHD enzymes are inhibited) and drive the expression of genes that increase iron availability. For example, HIF-2α in intestinal cells transcriptionally upregulates divalent metal transporter-1 (DMT-1), duodenal cytochrome b (DCYTB), and ferroportin to boost iron absorption, while systemically repressing hepcidin production [21]. More generally, the VHL–HIF pathway coordinately downregulates hepcidin and upregulates erythropoietin and ferroportin to mobilise stored iron for erythropoiesis [18]. HIFs also induce proteins (e.g., furin, TMPRSS6) that cleave hemojuvelin and blunt BMP signalling, further lowering hepcidin [19,39]. Thus, when iron or oxygen is scarce, HIF-mediated transcription shifts iron handling to favour uptake and release (via decreased hepcidin), ensuring that iron supply meets the increased demand for erythrocyte production and hypoxic adaptation [18,21].

## 3. Iron Metabolism and Homeostasis

The daily amount of iron absorbed from the diet (approximately 1–2 mg in adult males) is far less than the amount required to sustain these essential cellular and subcellular activities. To meet this demand, the body’s iron content is carefully regulated by proteins that homeostatically control cellular import, storage, and export [40,41]. Figure 2 summarises the import, storage, and export mechanisms of iron in different body cells.

### 3.1. Vascular Endothelial Cell Function and Control

Vascular endothelial cells line the lumen of all blood vessels and play key roles in maintaining vascular tone, anticoagulant, and immunomodulatory functions, and when required, haemostasis. The endothelium is not a simple barrier between blood and tissues; it is also an endocrine organ that can mediate organ function through a plethora of signalling pathways and cellular interactions. Numerous studies have shown that endothelial cells are involved in many pathological conditions, including cardiovascular diseases, inflammatory conditions and cancer. The function of ECs varies depending on their anatomical location, exposure to mechanical forces, and interactions with surrounding tissues. For example, arterial ECs experience high levels of shear stress and regulate vascular tone by releasing vasodilators. Conversely, venous ECs exposed to lower pressures play an essential role in immune cell trafficking and inflammation. The shape of ECs changes along the vascular network; however, in general, ECs are thin, usually hexagonal, and oriented longitudinally along the vessel axis. Their dimensions are approximately 50–70 µm long, 10–30 µm wide, and 0.1 to 1 µm thick [42]. The vascular endothelium is a semipermeable, selective barrier between the bloodstream and surrounding tissues that regulates the transport of various molecules into the interstitium and surrounding cells. As the continuous cellular interface forming the blood–tissue barrier, the vascular endothelium is uniquely and unremittingly exposed to circulating iron species—transferrin-bound iron, NTBI, cell-free haemoglobin, and free haem. It therefore occupies a privileged position with respect to iron compartmentalisation: it is at once a gatekeeper that must continuously regulate trans-endothelial iron flux and the first cell type to encounter decompartmentalised, redox-active iron once plasma scavenging capacity is exceeded. This dual role, combined with a high mitochondrial content and dependence on nitric oxide signalling that is readily quenched by free haem and haemoglobin, makes the endothelium a primary target of iron-mediated injury. Any disruption of the normal function of these cells can therefore result in adverse consequences, either acute or chronic, depending on the nature of the dysfunction. Detailed reviews of endothelial cell biology and function are available elsewhere [42,43].

### 3.2. Iron Compartmentalisation in the Blood and Vasculature

In the circulation, iron exists in distinct compartments. Nearly all plasma iron is bound to transferrin (no more than ~0.1% of total iron), while the vast majority is contained within erythrocytes (haemoglobin) and myocytes (myoglobin) [1]. Erythrocytes thus represent a mobile reservoir of iron, usually confined within the red cell’s protective environment. Intravascular haemolysis (as occurs in haemolytic anaemias, severe trauma, or extracorporeal circulation) ruptures this compartment: haemoglobin and haem are released into plasma, where they can overwhelm scavenger systems. Haptoglobin rapidly binds free haemoglobin, and hemopexin binds free haem, but the capacity of these systems is finite. When overloaded, unbound haemoglobin and haem accumulate and are potent oxidants [44]. Cell-free haemoglobin can undergo redox cycling, producing reactive oxygen species [44]. Indeed, in trauma and massive transfusion, haemolysis can exceed the binding capacity of haptoglobin/hemopexin, leading to pathological levels of cell-free haemoglobin. Correspondingly, experimental models show that haemolysis in trauma or sepsis releases sufficient haemoglobin to flood the plasma with redox-active iron, thereby mediating endothelial dysfunction and organ injury. Hemopexin is protective: mice lacking hemopexin develop renal injury upon haemolysis, underscoring the toxicity of free haem in vivo [6].

Erythrocyte-derived microparticles provide another source of labile iron in blood. Red-cell microparticles (microvesicles) shed under stress or during storage concentrate cell-free haem; for example, in sickle cell disease, up to one-third of circulating cell-free haem is carried within microparticles [45]. These haem-laden microvesicles can deliver haem directly to endothelial cells. Camus et al. showed that microparticle-bound haem is readily transferred to the endothelium, causing oxidative stress, endothelial activation, and microvascular occlusion [45]. Thus, haemolysis and RBC vesiculation produce a ‘cell-free’ iron pool that is highly pro-oxidant. When transferrin becomes saturated, non-transferrin-bound iron (NTBI) species appear in plasma—a heterogeneous mix of low-molecular-weight Fe^2+^ complexes that catalyse Fenton chemistry [46]. Proteins do not shield NTBI and readily generate hydroxyl radicals and other oxidants, exacerbating lipid peroxidation and oxidative damage in blood and vascular tissue [46].

Endothelial and other vascular cells take up this excess haem and iron, recompartmentalising it intracellularly. Endothelial cells internalise haemoglobin–haptoglobin complexes and free haem through endocytic mechanisms and induce haem oxygenase-1 to catabolise haem. Although the haemoglobin–haptoglobin scavenger receptor CD163 is the principal route for this uptake in monocytes and macrophages, its expression in vascular endothelium is low and remains a matter of debate. HO-1 enzymatically degrades haem to release ferrous iron, which is immediately sequestered by ferritin [47]. Indeed, exposed endothelium upregulates HO-1 and ferritin as a key defence: HO-1 cleaves the porphyrin ring (liberating iron), and ferritin then stores the liberated iron as ferric mineral within its shell [47]. These homeostatic adjustments protect the endothelium from haem-mediated injury [47]. However, when iron influx is excessive or sustained, ferritin stores can be overwhelmed. Sequestered (stored) iron then accumulates within endothelial lysosomes—an expansion of the storage compartment and a shift in intracellular iron distribution, rather than simply an increase in the labile pool—and eventually, as hemosiderin (an aggregated ferritin complex) in macrophages or perivascular cells, reflecting chronic iron overload [1].

Thus, pathological haemolysis and RBC breakdown lead to a maladaptive “re-compartmentalisation” of iron into vascular cells, where redox-active iron can persist. Clinically, such perturbations of iron compartmentalisation are seen in haemolytic anaemias (e.g., sickle cell disease, thalassemia, PNH), in major trauma with transfusions, and during extracorporeal circulation (cardiopulmonary bypass, ECMO) [48]. ARDS patients on ECMO, for instance, commonly develop intravascular haemolysis [48], and elevated cell-free haemoglobin levels are associated with worse outcomes. Similarly, severe trauma with haemorrhage or rhabdomyolysis releases haemoglobin/myoglobin that overwhelms scavengers. In these settings, the vascular endothelium is exposed to high “labile” iron–free haem and NTBI, which catalyse oxidative stress and inflammation. Such extracellular iron-catalysed reactions contribute to endothelial dysfunction: free haem amplifies leukocyte-mediated injury and promotes LDL oxidation [47], while NTBI and haemoglobin consume nitric oxide and oxidise lipids. Overall, abnormal iron trafficking in the blood—via haemolysis and microparticles—drives iron into the vascular wall, linking haemolytic/traumatic states to vascular oxidative injury and remodelling [45,48].

### 3.3. Intracellular Iron Loading and Organelle Dysfunction

Excess iron that enters vascular cells perturbs intracellular iron pools and organelles. Usually, cytosolic labile iron (Fe^2+^) concentration is kept very low; newly acquired iron is either immediately utilised (for haem/Fe–S synthesis) or stored safely in ferritin complexes. Ferritin (a 24-subunit sphere of H- and L-chains) oxidises Fe^2+^ to Fe^3+^ and mineralises it in its hollow core [49]. This ferroxidase activity of the ferritin heavy chain is essential to prevent Fenton chemistry: by sequestering iron as ferric oxyhydroxide, ferritin prevents iron from catalysing free radical formation [49]. Indeed, studies show that loss of ferritin heavy chain heightens susceptibility to oxidative stress and cell death upon iron loading [49]. However, if cellular iron uptake or release is dysregulated (for example, via hepcidin-mediated downregulation of ferroportin), the labile iron pool can expand beyond ferritin’s capacity. Elevated LIP promotes ROS generation in the cytosol, oxidising lipids and proteins and activating stress pathways. In vascular cells, this can promote pro-inflammatory signalling and DNA damage (analogous to findings in other systems where NTBI fosters oxidative stress [7].

Lysosomes are another critical iron depot. They accumulate iron through autophagy (ferritinophagy, mitophagy) or uptake of ferritin and senescent cell debris [50]. In an acidified lysosome, stored iron is released as Fe^3+^, then reduced to Fe^2+^, creating a labile lysosomal iron pool [50]. Under normal conditions, this Fe^2+^ is exported via channels (e.g., DMT-1, TRPML1) into the cytosol. However, excessive ferritinophagy (e.g., under chronic stress) can overload the lysosome: free Fe^2+^ accumulates, generating hydroxyl radicals and promoting lysosomal membrane damage [50]. Impaired lysosomal iron export (as seen in TRPML1 mutations) leads to iron-laden lysosomes and oxidative lipid–protein aggregates (lipofuscin), further compromising cell viability [50].

Mitochondria are central to cellular iron homeostasis and are profoundly affected by iron levels. Mitochondria import iron (via mitoferrins) to synthesise haem and Fe–S clusters for the electron transport chain [51]. A specialised mitochondrial ferritin also exists to buffer excess iron. When mitochondrial iron handling fails, organelle function falters: iron-overloaded mitochondria produce excessive reactive oxygen species (mtROS) and suffer membrane potential collapse [51]. In iron overload states, mitochondria accumulate DNA damage and respiratory chain defects. For example, excess iron exacerbates mitochondrial oxidative stress and lipid peroxidation, impairing mitochondrial membrane structure and function [51]. This mitochondrial dysfunction can impair ATP generation, increase mitochondrial apoptosis signals, and amplify cytosolic ROS. Conversely, mitochondrial iron deficiency (as seen in heart failure or BMPR2-linked defects) also compromises respiration and can trigger compensatory glycolysis. In pulmonary vascular cells, perturbations in mitochondrial iron—whether overload or relative insufficiency—thus derail energy metabolism and redox balance. Experimental studies further suggest that tissues differ substantially in their tolerance and transcriptional response to iron loading, with cardiac tissue appearing particularly sensitive to relatively small fluctuations in intracellular iron pools—presumably reflecting the narrow iron requirements necessary for optimal mitochondrial and contractile function [22].

Haemosiderin is a late-stage iron deposit visible under pathological conditions. It consists of aggregated ferritin and denatured proteins, as seen, for example, in macrophages after haemorrhage. Haemosiderin forms in chronically iron-loaded tissues when ferritin is degraded, and iron is slowly released, creating insoluble granules [52]. These haemosiderin-laden macrophages sequester iron to prevent excessive cytosolic free iron [52], but their presence also indicates prolonged iron dysregulation.

Aberrant intracellular iron and organelle iron can evoke vascular pathology. For instance, iron in atherosclerotic plaques can catalyse the oxidation of LDL, promoting foam cell formation and plaque growth [53]. Iron deposition is observed in hearts and kidneys in chronic overload disorders, where it drives mitochondrial dysfunction and fibrosis [51,53]. Ageing and neurodegenerative diseases likewise correlate with iron accumulation and oxidative damage [54]. In the vascular endothelium, excess iron can promote inflammation, reduce nitric oxide signalling, and induce senescence, contributing to hypertension and dysfunction. In hereditary haemorrhagic telangiectasia (HHT), chronic bleeding causes iron-deficiency anaemia, highlighting the flip side: iron scarcity may impair the vascular repair mechanism. Even therapeutic iron (e.g., intravenous iron in CKD) can transiently cause oxidative injury: certain IV iron formulations have been reported to induce tubular stress and proteinuria through oxidative mechanisms [55]. Iron also exerts dichotomous effects on cell fate: it is required for cell proliferation (tumour cells and fibroblasts upregulate iron uptake to drive DNA synthesis [56], yet excess iron primes cells for ferroptosis—an iron-dependent form of cell death [57].

## 4. Clinical and Disease Settings with Implications for Dysregulated Iron Homeostasis

A substantial and growing body of literature demonstrates a role for disrupted regulatory control of iron resources in an array of diseases and morbidities involving the vascular compartment, including vascular dementia, kidney disease (acute and chronic) and cardiovascular diseases such as chronic heart failure and atherosclerosis [58].

Genetic diseases—including thalassaemia, sickle cell disease, hereditary haemorrhagic telangiectasia (HHT), and other disorders of deficient haemoglobin production and/or haemolysis—can be associated with dysregulated iron homeostasis [59]. Decompartmentalisation of red-cell iron-containing components, including haemoglobin and its breakdown products haem and iron, poses a significant risk to the function of vascular endothelial cells once endogenous protection is saturated. Hereditary haemochromatosis (a series of genetic disorders) results in the excessive accumulation of tissue iron stores, including in the vasculature [59].

Conditions associated with chronic hypoxaemia are characterised by secondary erythrocytosis, increased haemoglobin production, and increased iron demand and altered iron homeostasis. As an example, Eisenmenger syndrome, a congenital disease of the heart involving intra- or extracardiac shunting of blood, leads to pulmonary arterial hypertension, chronic hypoxaemia, and many end-organ manifestations of low oxygen levels [60]. Iron deficiency is a frequently observed feature of this condition.

Surgical interventions involving extracorporeal circuits, such as cardiopulmonary bypass (CPB), can lead to haemolytic events, as can the return of salvaged blood, retrieved via suction, to the patient, which is also a common practice in trauma and thoracic surgeries of longer duration [61]. These haemolytic events can contribute to the activation of vascular endothelial cells, interactions between inflammatory cells, and the upregulation of clotting cascades, resulting in detrimental blood clot formation if anticoagulation protocols fail [62]. Organ damage, including acute kidney injury (AKI), is an often-seen adverse complication from such surgeries, and a growing body of evidence has linked this to haemolytic events. The use of extracorporeal devices for patients requiring heart and/or lung support of longer duration, such as extracorporeal membrane oxygenation (ECMO) or ventricular support devices (VADs) to bridge patients to transplant, is becoming more mainstream, with similar potential implications to those seen with CPB. In these settings, intravascular haemolysis represents a direct breakdown of systemic iron compartmentalisation, exposing the vascular endothelium to cell-free haemoglobin, haem, and non-transferrin-bound iron. Red cell blood transfusion has similarly been shown to contribute to a haemolytic load, with evidence suggesting links to acute lung injury (transfusion-related lung injury, TRALI) and AKI in some critically ill patients [63]. Even the use of iron supplements can have adverse consequences in specific settings [64].

The consequences of disrupted iron control and decompartmentalisation are numerous. In vascular endothelial cells, these include Toll-like receptor (TLR) activation, intracellular iron accumulation, ROS-mediated stress signalling, endothelial activation, and the development of a pro-inflammatory phenotype. Table 1 lists the relevant disease processes and consequences for vascular endothelial cells, as discussed above, along with the cited literature. The remainder of the review focuses on a more in-depth exploration of endothelial cell dysfunction in pulmonary hypertension.

### 4.1. Pulmonary Hypertension: A Case in Point

Pulmonary hypertension (PH) is a haemodynamic and pathophysiological state defined as a mean pulmonary artery pressure > 20 mmHg at rest, measured by right heart catheterisation [89]. It is further classified into 5 groups based on underlying pathophysiology and anticipated treatment response. One of the hallmarks of PH is pulmonary vascular remodelling, resulting in increased pulmonary vascular resistance, and ultimately, right heart failure [90]. This is associated with high morbidity and mortality, necessitating a deeper understanding of its underlying mechanisms to identify novel targets for intervention. Although characteristically associated with Group 1; pulmonary arterial hypertension (PAH), pulmonary vascular remodelling can be seen in all groups and is usually associated with severity and poor outcome [90].

In recent years, iron homeostasis has emerged as a key factor in the pathogenesis of pulmonary vascular remodelling and the development of PH [91]. Iron is essential for cellular respiration, oxidative stress regulation, and vascular function. Dysregulated iron metabolism, particularly the mis-compartmentalisation of iron within endothelial and smooth muscle cells, has been implicated in pulmonary vascular remodelling [92]. Notably, the hepcidin–ferroportin axis, a critical regulatory pathway governing iron transport and storage, has been shown to influence vascular remodelling and mitochondrial function in pulmonary vascular cells [32,93,94].

**Figure 3:**
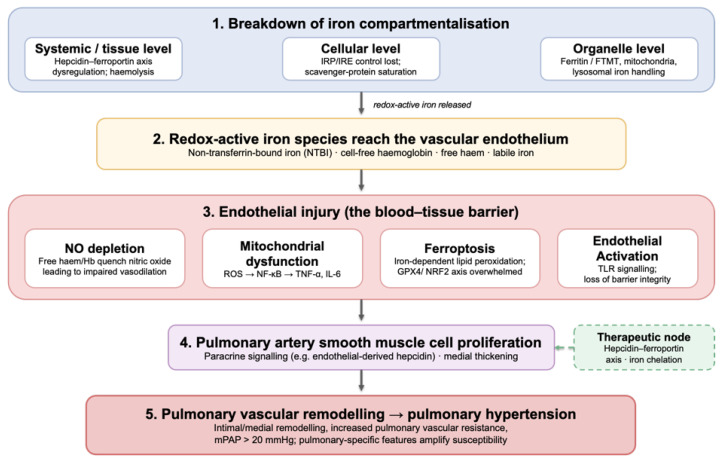
Breakdown of iron compartmentalisation at the systemic/tissue, cellular, and organelle levels (1) expands redox-active iron pools and promotes the appearance of non-transferrin-bound iron, cell-free haemoglobin, and free haem, which reach the vascular endothelium (2). At the blood–tissue barrier, these species drive endothelial injury (3) through nitric oxide depletion, mitochondrial dysfunction with NF-κB-mediated inflammatory signalling, ferroptotic cell death, and endothelial activation. The resulting paracrine signalling promotes pulmonary artery smooth muscle cell proliferation (4), culminating in pulmonary vascular remodelling and pulmonary hypertension (5). Pulmonary-specific haemodynamic and signalling features amplify this susceptibility. The hepcidin–ferroportin axis (green) represents a principal regulatory node and potential therapeutic target. FTMT, mitochondrial ferritin; GPX4, glutathione peroxidase 4; IRP/IRE, iron regulatory protein/iron-responsive element; mPAP, mean pulmonary arterial pressure; NRF2, nuclear factor erythroid 2-related factor 2; TLR, Toll-like receptor.

Several features distinguish the pulmonary circulation from systemic vascular beds and may render it particularly vulnerable to disordered iron handling. It is a low-pressure, high-compliance, high-flow circuit that receives the entire cardiac output and is exposed to the highest oxygen tensions in the body, favouring iron-catalysed reactive oxygen species generation across an exceptionally thin gas-exchange barrier. Uniquely, the pulmonary vasculature constricts rather than dilates in response to hypoxia (hypoxic pulmonary vasoconstriction), so the HIF–iron axis that couples oxygen sensing to iron handling carries distinct haemodynamic consequences here. Pulmonary arterial endothelial and smooth muscle cells also sit at the intersection of iron regulation and BMPR2/BMP signalling, the pathway most frequently disrupted in heritable pulmonary arterial hypertension, and IL-6–hepcidin signalling within this bed links inflammation directly to local iron retention. These pulmonary-specific characteristics, rather than the mere occurrence of pulmonary hypertension, are the reason iron compartmentalisation is examined here using the pulmonary circulation as the principal example. Despite growing recognition of iron’s role in PH, significant gaps remain in our understanding of how iron compartmentalisation contributes to endothelial dysfunction and smooth muscle cell proliferation—two hallmarks of pulmonary vascular remodelling. The following sections synthesise current evidence on the intersection of iron metabolism and vascular dysfunction in PH, with a particular focus on endothelial cell biology, mitochondrial dysregulation, and smooth muscle remodelling, highlighting potential therapeutic avenues and key research directions. The integrated mechanism by which disrupted iron compartmentalisation drives endothelial injury and pulmonary vascular remodelling is summarised in Figure 3.

#### 4.1.1. Mitochondrial Function in Pulmonary Hypertension

Recent findings have revealed significant insights into the relationship between the hepcidin–ferroportin axis, mitochondrial function, and vascular cell behaviour in pulmonary hypertension. The mitochondrion is the primary organelle mediating complex metabolic pathways in bioenergetics, biosynthesis, and cell signalling. Disruption of mitochondrial function can lead to altered cellular phenotypes that contribute to pathological conditions such as PH [95].

Mitochondrial iron regulation is critical for maintaining cellular homeostasis [93], including overall cellular iron balance. Mitochondria import cytosolic iron for cofactor assembly, but they also control reactive iron via their own ferritin [96]. Disruption of mitochondrial iron handling (for example, by hepcidin-triggered ferroportin loss) leads to mitochondrial iron loading and dysfunction [49,51]. Excess mitochondrial iron impairs the electron transport chain and elevates mtROS production [51]. For instance, loss of ferritin heavy chain (which oxidises and sequesters Fe^2+^ in the cytosol—mitochondrial iron is instead buffered by the chemically and genetically distinct mitochondrial ferritin, FTMT) makes cells far more susceptible to iron-induced oxidative stress [49]. Such mitochondrial iron dysregulation leads to bioenergetic failure and can activate pro-growth signalling. Hepcidin-mediated ferroportin degradation promotes intracellular iron retention, which may secondarily increase mitochondrial iron accumulation and ROS generation. The resulting mitochondrial impairment can contribute to the metabolic and functional abnormalities seen in pulmonary hypertension, linking mitochondrial iron to disease.

Mitochondrial dysfunction is a hallmark of PH pathogenesis. A characteristic finding is a shift to glycolytic metabolism (“Warburg effect”): PAECs and PASMCs from PAH patients show stabilisation of HIF-1α/2α and increased PDK expression, driving aerobic glycolysis over oxidative phosphorylation [97]. Mitochondrial biogenesis and mtDNA replication are also impaired in PH, leading to fewer, less functional mitochondria [97]. At the same time, mitochondrial reactive oxygen species (mtROS) are elevated in PH lesions, as evidenced by increased oxidative markers (e.g., 8-OHdG) and redox-sensitive signalling [97]. The excess ROS act as signalling molecules: they activate NF-κB signalling, which in turn drives the production of inflammatory cytokines (TNF-α, IL-6), which further suppress mitochondrial function and drive vascular inflammation [97]. Moreover, genetic defects affecting mitochondrial iron–sulphur biogenesis (such as NFU1 mutations) impair Complex II activity and are linked to PAH, underscoring the importance of mitochondrial iron handling in this disease [97]. In summary, PH involves a vicious cycle of mitochondrial defects: metabolic reprogramming and oxidative stress reinforce each other, promoting PASMC proliferation and endothelial dysregulation [97].

Studies by this group have demonstrated that pulmonary artery endothelial cells (PAECs) express ferroportin, and hepcidin treatment results in mitochondrial iron accumulation and intracellular hepcidin biosynthesis and release [94]. The findings show striking differences in mitochondrial responses between PAECs and PASMCs upon hepcidin exposure, with PASMCs exhibiting more pronounced mitochondrial dysfunction. This implies that PAECs are more tolerant of hepcidin exposure than PASMCs, as evidenced by minimal changes in mitochondrial morphology, ROS production, and respiration. The differential response to hepcidin between these cell types may have important implications for understanding the pathophysiology of pulmonary hypertension and developing targeted therapeutic approaches.

#### 4.1.2. Iron Compartmentalisation and Cell Migration/Proliferation in Pulmonary Hypertension

Disruption of iron compartmentalisation within cells can alter cellular functions, including migration and proliferation. Earlier work by this group demonstrated that conditioned media from hepcidin-treated PAECs induced significant changes in PASMCs, including loss of ferroportin expression, increased migration, and enhanced proliferation [94].

When PASMCs were incubated with conditioned media from PAECs treated with hepcidin, there was almost complete loss of ferroportin staining as determined by confocal imaging. This effect was not observed with media from untreated PAECs or with “hepcidin in media only” controls, suggesting that an endothelial-derived component, possibly hepcidin, was responsible for this change [32,94]. Conditioned media from IL-6- or hepcidin-challenged PAECs induced significant PASMC migration, with concentration-dependent increases in migratory response. Conditioned media analysis revealed substantial increases in hepcidin and cytokines, suggesting a paracrine effect of hepcidin that may feed into remodelling in the pulmonary artery [94,98].

Iron availability powerfully influences the behaviour of pulmonary endothelial and smooth muscle cells. Experimental studies show that reducing iron blunts vascular remodelling: for example, the iron chelator deferoxamine prevents hypoxia-induced PH in rats and directly inhibits PASMC growth in culture [99]. Similarly, plumbagin—a plant-derived iron chelator—suppresses human PASMC proliferation and reduces arterial muscularisation in PAH models [41]. Conversely, excess iron has been shown to promote vascular cell proliferation in several experimental models. Gorbunov et al. reported that adding iron to cultured PAECs induces proliferation and pro-remodelling changes [41]. At the molecular level, cytokines that alter iron handling affect growth: IL-6 (which raises hepcidin) strongly enhances PASMC proliferation [41]. This is consistent with the observation that PAECs and PASMCs both express ferroportin, and that hepcidin-induced ferroportin internalisation leads to intracellular iron retention and cell growth [41].

In contrast, iron chelation or deficiency suppresses these processes. These findings suggest that hepcidin can induce PAECs to release factors that promote PASMC migration and proliferation, potentially contributing to the vascular remodelling observed in pulmonary hypertension. The intimate relationship between iron compartmentalisation, mitochondrial function, and cellular behaviour provides new insights into the mechanisms underlying pulmonary vascular pathology.

#### 4.1.3. Ferroptosis in Pulmonary Hypertension

Ferroptosis is a form of regulated cell death distinct from apoptosis, necrosis, and autophagy, characterised by iron-dependent lipid peroxidation and extensive membrane lipid damage and membrane destabilisation [100]. Unlike apoptosis, which relies on caspase activation, ferroptosis is driven by an accumulation of lipid hydroperoxides due to impaired glutathione peroxidase 4 (GPX4) activity and increased intracellular iron. Given the central role of iron metabolism in pulmonary hypertension (PH), ferroptosis has emerged as a phenotype of endothelial dysfunction and vascular remodelling [101].

#### 4.1.4. Iron Dysregulation and Ferroptosis in Vascular Cells

Iron overload and mis-compartmentalisation within pulmonary vascular cells may promote ferroptotic cell death, contributing to endothelial damage and loss of vascular integrity. PAECs are particularly susceptible to oxidative stress, and perturbations in the hepcidin–ferroportin axis may exacerbate lipid peroxidation and ferroptotic pathways. Studies have demonstrated that iron accumulation in vascular cells can trigger ferroptosis, contributing to endothelial damage and loss of vascular integrity; iron dysregulation is, separately, associated with smooth muscle proliferation, and together, these processes constitute the hallmarks of PH pathology [101,102].

In contrast to PAECs, PASMCs exhibit resistance to ferroptosis under certain conditions, potentially favouring their hyperproliferative phenotype in PH [103]. Ferroportin downregulation in PASMCs may contribute to intracellular iron retention, facilitating redox cycling and lipid peroxidation [32]. These variations in cell-type responses to mitochondrial and iron overload may drive pathology in pulmonary vascular remodelling. However, whether ferroptosis is a driver or consequence of its pathogenesis remains an open question.

#### 4.1.5. Glutathione Metabolism and Ferroptosis Sensitivity in PH

Glutathione (GSH) depletion and impaired GPX4 activity are critical determinants of ferroptosis sensitivity. PAH patients often exhibit increased oxidative stress, which may arise from dysregulated GSH metabolism [99]. The interplay between iron accumulation and glutathione depletion suggests that ferroptosis may contribute to the progressive endothelial damage observed in PH. Recent evidence has implicated NRF2, a master regulator of antioxidant defence, in modulating ferroptosis sensitivity. NRF2 activation in PH may act as a compensatory mechanism to mitigate ferroptotic stress, but its dysregulation could also exacerbate vascular pathology [104].

A critical determinant of ferroptosis sensitivity is the glutathione (GSH) antioxidant system. GSH is synthesised from cysteine (via the γ-glutamyl cycle) and serves as the cofactor for glutathione peroxidase-4 (GPX4). SLC7A11 imports cystine for GSH synthesis, while the enzyme glutamate–cysteine ligase (GCLC) drives its production [13]. GPX4 then uses GSH to reduce lipid hydroperoxides to harmless lipid alcohols. Thus, intact cystine/GSH/GPX4 activity is essential for neutralising iron-catalysed lipid peroxidation [69]. In the context of PH, oxidative stress and inflammation may deplete GSH or impair these enzymes, predisposing endothelial cells to ferroptosis. Notably, nuclear factor erythroid 2-related factor 2 (NRF2) is the master regulator of this defence [13,69]. NRF2 drives the transcription of SLC7A11, GCLC, GPX4, and NADPH-regenerating enzymes, as well as ferritin and ferroportin. Through these targets, NRF2 ensures an ample supply of GSH and limits free iron. If NRF2 signalling is compromised (as may occur under chronic oxidative stress), cells lose this protection. Indeed, increased levels of ferroptosis markers have been observed when NRF2 is inhibited [13,69]. Consistent with this, experimental iron loading in vivo elicits coordinated transcriptional responses within the NRF2-regulated antioxidant network, with NRF2 (Nfe2l2) expression correlating with that of GPX4, SOD isoforms, and nitric oxide synthases in iron-handling tissues, providing further mechanistic context for the link between iron accumulation and redox imbalance [22].

Multiple pathways thus intersect in PH: impaired SLC7A11 function or cysteine availability, reduced GPX4 activity, and excessive iron combine to trigger lipid peroxidation. In vitro, inhibiting GPX4 or depleting GSH in pulmonary endothelial cells causes ferroptotic death, which iron chelators or lipophilic antioxidants can rescue [105]. Conversely, activating NRF2 or supplementing GSH precursors protects against ferroptosis [106]. In experimental PH, agents that upregulate NRF2/GPX4 signalling (for instance, dapagliflozin or other NRF2 activators) have been shown to mitigate vascular remodelling by limiting endothelial ferroptosis. Thus, dysregulation of glutathione metabolism—whether due to oxidative damage, nutrient deficiency, or disrupted NRF2 signalling—may sensitise pulmonary vascular cells to ferroptosis. This links iron overload (or misdistribution) directly to a regulated cell death pathway that underlies endothelial injury and inflammation in PH.

#### 4.1.6. Potential Therapeutic Targeting of Ferroptosis in PH

Given the emerging role of ferroptosis in PH, therapeutic strategies targeting ferroptotic pathways are of increasing interest. Ferroptosis inhibitors, such as ferrostatins and liproxstatins, have shown promise in preclinical models of cardiovascular disease by preventing lipid peroxidation and cell death [107]. Additionally, modulation of the hepcidin–ferroportin axis to correct iron homeostasis may mitigate ferroptotic cell death and thereby influence pulmonary vascular remodelling.

Further studies are required to elucidate the precise role of ferroptosis in PH pathophysiology and to determine whether ferroptosis-targeting interventions could be integrated into existing treatment regimens. Understanding the balance between ferroptotic susceptibility and resistance in different vascular cell populations may provide new insights into disease progression and therapy.

#### 4.1.7. Therapeutic Perspective on Pulmonary Hypertension

Clinical studies have investigated the potential therapeutic benefits of iron supplementation in patients with PAH. A pilot study by Viethen et al. (2014) showed that intravenous iron supplementation improved exercise capacity, quality of life, and right ventricular function in patients with PAH and iron deficiency [108]. Similarly, Ruiter et al. (2015) reported improvements in exercise capacity and quality of life after iron supplementation in patients with idiopathic PAH [109]. In addition, Tay et al. demonstrated improvement in exercise capacity and quality of life in patients with Eisenmenger syndrome who were given 3 months of iron replacement [110]. Moreover, a recent systematic review of iron supplementation in PAH patients concludes that positive effects were observed in patients regardless of study design [111]. However, despite encouraging findings from several smaller and observational studies, results from controlled interventional trials have been less consistent. For example, a double-blind, placebo controlled, cross-over study, in patients with PAH who were iron deficient, but not anaemic, demonstrated no clinical benefit of a slow-release iron preparation as a single infusion (apart from improving iron status) [112].

Therefore, the therapeutic approach to iron dysregulation in PAH remains challenging. While iron supplementation may benefit some patients, it could exacerbate the disease in others, depending on the underlying mechanisms. For example, in patients with elevated hepcidin levels, iron supplementation may not effectively correct iron deficiency due to impaired iron absorption and mobilisation. Furthermore, excessive iron supplementation could lead to iron overload and increased oxidative stress, potentially worsening vascular remodelling and right ventricular dysfunction.

Recent advances in our understanding of the hepcidin–ferroportin axis and its role in pulmonary vascular remodelling have led to the exploration of novel therapeutic approaches targeting this pathway. Inhibition of hepcidin production or activity, or stabilisation of ferroportin expression, could restore iron homeostasis and potentially ameliorate pulmonary vascular remodelling in PAH. The monoclonal antibody LY2928057, which stabilises ferroportin membrane localisation and activity, has shown promise in preclinical studies [32] and could represent a potential therapeutic option for patients with PAH.

In summary, emerging evidence links abnormal iron compartmentalisation and mitochondrial dysfunction to vascular pathology in PH. Disruption of normal iron compartments delivers catalytic iron to the endothelium, promoting oxidative damage and inflammation. Within cells, excess iron accumulates, altering metabolism and redox balance, leading to endothelial dysfunction and smooth muscle proliferation characteristic of pulmonary vascular remodelling. Finally, overloaded vascular cells undergo ferroptotic injury, a process sensitive to GPX4 and NRF2 defence mechanisms. Thus, perturbations in iron handling—at the level of blood, organelles and antioxidant pathways—converge to drive the vascular dysfunction of PH.

## 5. Conclusions

Iron is essential for aerobic life; it is a finite resource that is largely recycled to compensate for limited dietary uptake capacity. Strict control of iron mobilisation is essential to prevent the unregulated catalysis of damaging ROS production and to restrict microbial access to iron and thereby limit pathogen growth. Loss of such regulatory controls can additionally lead to the saturation of specific antioxidant protection and altered compartmentalisation of iron resources within cellular environments impacting cell fate. Vascular endothelial cells are susceptible to such events, particularly so because they are interfaced to the blood supply, which is rich in iron species, either bound to iron- and haem-binding plasma proteins, within red cells, or as free species in adverse circumstances. An array of disease processes, some surgical procedures, and supportive therapeutic approaches can alter balanced iron mobilisation and control, affecting endothelial cell function and fate with potential detrimental consequences. Of particular relevance is the pulmonary vasculature, where dysregulated iron compartmentalisation contributes to the endothelial dysfunction, mitochondrial impairment, and smooth muscle cell proliferation that are central to pulmonary vascular remodelling and the progression of pulmonary hypertension. Correcting disordered iron handling therefore represents a compelling, if complex, therapeutic target in this disease.

## Figures and Tables

**Table 1:** Disease states and clinical scenarios associated with dysregulated iron homeostasis and their consequences for vascular endothelial cell function. For each condition, the principal mechanism of iron dysregulation or decompartmentalisation is summarised alongside the resulting endothelial pathobiology. Conditions are grouped broadly to reflect shared mechanisms: intrinsic haemolytic and genetic disorders (atherosclerosis, sickle cell disease, thalassaemia, Eisenmenger syndrome), acquired haemolytic states arising from extracorporeal circuit use or transfusion, and iatrogenic iron loading from supplementation. In each setting, loss of normal iron compartmentalisation —whether through intravascular haemolysis, saturation of scavenger proteins (haptoglobin, hemopexin), accumulation of non-transferrin-bound iron (NTBI), or ferroportin-mediated iron retention—exposes the vascular endothelium to redox-active iron species. The downstream consequences include nitric oxide (NO) depletion, oxidative stress, endothelial activation, increased expression of adhesion molecules, and in chronic settings, vascular remodelling. AKI, acute kidney injury; BBB, blood–brain barrier; CPB, cardiopulmonary bypass; ECMO, extracorporeal membrane oxygenation; ER, endoplasmic reticulum; Hb, haemoglobin; HO-1, haem oxygenase-1; NTBI, non-transferrin-bound iron; NO, nitric oxide; RBC, red blood cell; VAD, ventricular assist device.

Disease or Morbidity	Overall Presentation	Implications for Vascular Endothelial Cells	Key References
**Atherosclerosis**	Intraplaque haemorrhage introduces erythrocyte debris & free haem into the plaque environment, driving oxidative and inflammatory progression.	Free haem and cell-free Hb induce oxidative stress, ER stress, lipid oxidation and cytotoxicity in endothelial cells, promoting endothelial dysfunction & plaque instability. HO-1/HO-2 pathways are protective but can be overwhelmed.	[65,66,67]
**Vascular dementia/cerebral small vessel disease**	Progressive cognitive decline associated with microvascular damage, microbleeds & BBB dysfunction, often with iron deposition & oxidative stress.	Iron accumulation and haem products impair BBB integrity, increase endothelial permeability, promote inflammation and disrupt cerebrovascular autoregulation, contributing to neurodegeneration.	[68,69,70,71]
**Eisenmenger disease**	Long-term cyanotic congenital heart disease with hypoxaemia, erythrocytosis, and haemolysis-associated complications.	Chronic haemolysis increases plasma free haemoglobin, reducing NO bioavailability and impairing vasodilation. Endothelial dysfunction contributes to pulmonary hypertension, thrombosis, and systemic vasculopathy.	[72,73,74]
**Sickle cell disease & thalassaemia**	Inherited haemolytic anaemias characterised by chronic intravascular haemolysis, erythrocyte rigidity, and vascular occlusion; iron dysregulation is compounded by repeated transfusions.	Chronic haemolysis saturates haptoglobin and hemopexin, releasing cell-free Hb and haem that deplete NO, promote oxidative stress, and activate the endothelium; microparticle-bound haem is directly transferred to endothelial cells, driving vascular occlusion and dysfunction.	[45,59,75]
**Cardiac surgery with cardiopulmonary bypass (CPB)**	Mechanical trauma to RBCs during CPB causes acute haemolysis, raising circulating free haemoglobin and iron. Often associated with postoperative vasoplegia and AKI.	Cell-free Hb scavengers NO, causes oxidative stress, and directly injures the endothelium, leading to vasomotor dysfunction, increased permeability and microvascular injury. High free Hb correlates with worse outcomes.	[76,77,78,79,80]
**Other extracorporeal devices (ECMO, VADs, dialysis circuits)**	High shear stress causes ongoing haemolysis; plasma free Hb levels can become markedly elevated. Associated with inflammation, thrombosis, and multi-organ injury.	Free Hb and haem activate endothelium, increase permeability, induce oxidative stress, and promote microvascular dysfunction. Linked to AKI, coagulopathy and mortality.	[61,76,81,82]
**Transfusion & haemolytic disease processes.**	Chronic haemolysis with increased circulating haemoglobin, haem and iron; high oxidative burden and systemic inflammatory activation.	Endothelial activation, increased adhesion molecules, oxidative stress, and NO depletion promote vasculopathy, thrombosis, and microvascular obstruction.	[75,83,84]
**Iron supplements (oral or IV)**	Used to correct iron deficiency; IV iron transiently increases NTBI and affects redox balance, especially in CKD or HF patients.	High-dose IV iron can modulate endothelial activation markers (E-selectin, P-selectin) and oxidative stress, though effects vary by formulation and patient population; oral iron generally has minimal acute endothelial impact.	[85,86,87,88]

## Data Availability

No new data were created or analysed in this study.
